# Supplementary material for: Prevalence of musculoskeletal injuries among university undergraduates following Sri Lankan traditional dancing
Source: PLoS One. 2023 Aug 10;18(8):e0288155. doi: 10.1371/journal.pone.0288155 (PMC10414596; doi:10.1371/journal.pone.0288155)
Supplement: S1 File — (DOCX) [file pone.0288155.s001.docx]

**Supporting information: Questionnaire**

This questionnaire is given to you to identify musculoskeletal injuries among Sri Lankan traditional dancers**.** Please be kind enough to provide genuine answers to the questions by yourself. Your privacy will be maintained. This information will be kept highly confidential and will only be used for the research purpose. Your participation is highly appreciated.

**PART I- Identification**

1. Age:
2. Gender:

- Female
- Male

1. Ethnicity:
2. Height (cm):
3. Weight (kg):
4. University/ Institute:
5. Academic year:

- 1^st^ year
- 2^nd^ year
- 3^rd^ year
- 4^th^ year

1. Have you undergone any surgery during the past 2 year?

Yes

No

(a). If yes, was it done because of an injury occurred during dancing?

1. Do you have any joint/muscle abnormality for long period of time?

Yes

No

If yes,

1. Where and what type of abnormality is it? ………………………………………………..

**Part II- Aspects related to dance**

1. Age when you started dancing:
2. Which dance type/types that you practice currently:

- Sri Lankan traditional dance
- Western dance
- Indian traditional dance
- Other

1. If you are following Sri Lankan traditional dancing what is the,

(a). main style

- Kandyan
- *Sabaragamu*
- Low country

(b). Sub style

- Kandyan
- Low country
- *Sabaragamu*

1. Are you doing any exercises prior to the dancing or dancing practice? (stretching/warm up)

- Yes
- No

1. If “**yes”**,

- Stretching exercise -: Duration ___________min
- Warm up -: Duration ___________min

1. Are you doing any exercises after the dancing or dancing practices?

- Yes
- No

1. If “**yes”**,

- Stretching exercise -: Duration ___________min
- Cool Down -: Duration ___________min

1. Duration of Practices-

- Hours per day ___________
- Days per week ___________

1. Kind of Practice:

- Vigorous / high speed
- Moderate speed
- Low speed

1. What is the dance style that you feel more tired when you practicing,

- Kandyan
- Low country
- *Sabaragamu*

1. How long do you practice per session? ________ hrs
2. How many intervals do you take during one session? ________________
3. On what type of a surface do you practice dancing?

- Cement floor
- Tile floor
- Sand floor

1. Do you practice any other physical activity in regular basis: member in a sports team?
   - Yes
   - No
2. If “**Yes**”,

- Vigorous sport activity (eg: volley ball, Swimming) _____________
- Gym _____________
- Yoga _____________
- Other _____________

1. If “**yes**” (24),
2. Specify

Days per week _____________

Hours per day _____________

1. How long have you practiced this activity? _____________

**Part III- Aspects related to injury.**

1. Have you ever suffered any injury?

- Yes
- No

If yes, which one from the below?

**Right**

**Left**

- - Fracture / separation of bones Location ________ ______

or crack on bones

- - Strain / Location ________ ______

Muscle rupture

- - Dislocation / Location ________ ______

joint separation

- - Subluxation / Location ________ ______

half separation of joints

- - Sprain / Location ________ ______

ligament rupture

- - Others Location ________ ______

1. If “**yes”**, how long ago?

- 6 months
- 6 months - 1 year
- 1 - 5 years
- Over 5 years

1. How did the injury happen during the dance?

- Prolong *mandiya* position
- Backward handspring (Back flips)
- Acrobatics (handspring)
- Leaps (jump long way)
- Twirls (spin quickly)
- Jumps
- Other

1. According to the Body chart below, select the location of injuries:


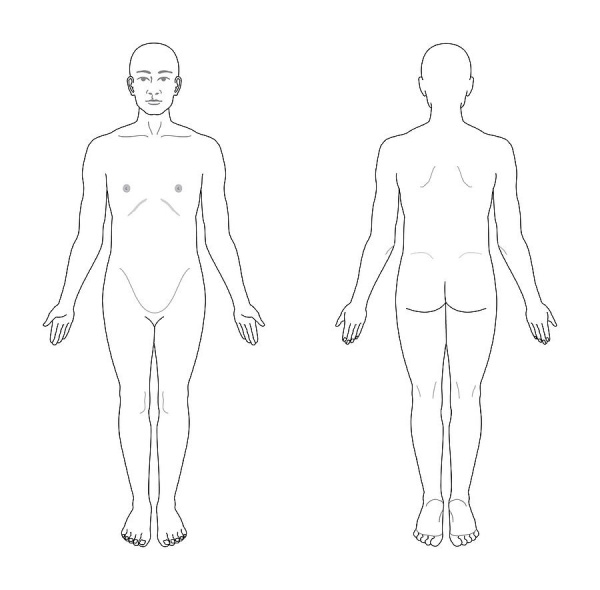


29. Have you had any kind of treatment for this injury?

- Yes
- No

30. Have you had any kind of physiotherapy for this lesion?

- Yes
- No

1. For which one?

_____________________________________________________________________________

1. For how long?

_____________________________________________________________________________

31. After injury, how long you were away from dancing?

______________________________________________________________________

32. Do you feel any kind of pain during dance practice? Select the location of the pain from the chart and mark the severity of your pain. (0- no pain, 1-3 apparent pain,4-6 moderate pain,7-9 severe pain,10 unbearable)

| Face | 0 | 1 | 2 | 3 | 4 | 5 | 6 | 7 | 8 | 9 | 10 |
| --- | --- | --- | --- | --- | --- | --- | --- | --- | --- | --- | --- |
| Neck | 0 | 1 | 2 | 3 | 4 | 5 | 6 | 7 | 8 | 9 | 10 |
| Abdomen | 0 | 1 | 2 | 3 | 4 | 5 | 6 | 7 | 8 | 9 | 10 |
| Right shoulder/Upper chest. | 0 | 1 | 2 | 3 | 4 | 5 | 6 | 7 | 8 | 9 | 10 |
| Left shoulder/ Upper Chest | 0 | 1 | 2 | 3 | 4 | 5 | 6 | 7 | 8 | 9 | 10 |
| Right elbow | 0 | 1 | 2 | 3 | 4 | 5 | 6 | 7 | 8 | 9 | 10 |
| Left elbow | 0 | 1 | 2 | 3 | 4 | 5 | 6 | 7 | 8 | 9 | 10 |
| Right forearm | 0 | 1 | 2 | 3 | 4 | 5 | 6 | 7 | 8 | 9 | 10 |
| Left forearm | 0 | 1 | 2 | 3 | 4 | 5 | 6 | 7 | 8 | 9 | 10 |
| Hand/right fist | 0 | 1 | 2 | 3 | 4 | 5 | 6 | 7 | 8 | 9 | 10 |
| Hand/left fist | 0 | 1 | 2 | 3 | 4 | 5 | 6 | 7 | 8 | 9 | 10 |
| Lumbar spine | 0 | 1 | 2 | 3 | 4 | 5 | 6 | 7 | 8 | 9 | 10 |
| Pelvic region | 0 | 1 | 2 | 3 | 4 | 5 | 6 | 7 | 8 | 9 | 10 |
| Buttocks | 0 | 1 | 2 | 3 | 4 | 5 | 6 | 7 | 8 | 9 | 10 |
| Hip/right thigh | 0 | 1 | 2 | 3 | 4 | 5 | 6 | 7 | 8 | 9 | 10 |
| Hip/left thigh | 0 | 1 | 2 | 3 | 4 | 5 | 6 | 7 | 8 | 9 | 10 |
| Right Knee | 0 | 1 | 2 | 3 | 4 | 5 | 6 | 7 | 8 | 9 | 10 |
| Left knee | 0 | 1 | 2 | 3 | 4 | 5 | 6 | 7 | 8 | 9 | 10 |
| Right leg | 0 | 1 | 2 | 3 | 4 | 5 | 6 | 7 | 8 | 9 | 10 |
| Left leg | 0 | 1 | 2 | 3 | 4 | 5 | 6 | 7 | 8 | 9 | 10 |
| Right ankle | 0 | 1 | 2 | 3 | 4 | 5 | 6 | 7 | 8 | 9 | 10 |
| Left ankle | 0 | 1 | 2 | 3 | 4 | 5 | 6 | 7 | 8 | 9 | 10 |
| Right foot | 0 | 1 | 2 | 3 | 4 | 5 | 6 | 7 | 8 | 9 | 10 |
| Left foot | 0 | 1 | 2 | 3 | 4 | 5 | 6 | 7 | 8 | 9 | 10 |

33. What are the suggestions do you have to prevent dance related injuries.
