# Supplementary material for: Prevalence of musculoskeletal injuries among university undergraduates following Sri Lankan traditional dancing
Source: PLoS One. 2023 Aug 10;18(8):e0288155. doi: 10.1371/journal.pone.0288155 (PMC10414596; doi:10.1371/journal.pone.0288155)
Supplement: Attachment — Submitted filename: PONE-D-22-35570.pdf [file pone.0288155.s002.pdf]

PLOS ONE

Prevalence of musculoskeletal injuries among university undergraduates following Sri Lankan traditional dancing

--Manuscript Draft--

**Manuscript Number:**	PONE-D-22-35570
**Article Type:**	Research Article
**Full Title:**	Prevalence of musculoskeletal injuries among university undergraduates following Sri Lankan traditional dancing
**Short Title:**	Musculoskeletal injuries in Sri Lankan traditional dancing
**Corresponding Author:**	Surangika Imanthi Wadugodapitiya, Ph.D<br>University of Peradeniya<br>Peradeniya, Central SRI LANKA
**Keywords:**	injury mechanism; musculoskeletal injuries; prevalence; Sri Lankan traditional dancing; university undergraduates
**Abstract:**	Dancing is a demanding form of art that consists of a purposefully selected series of human movements presented in a rhythmic way. However, Dancers represent a medically under-served occupational group who are at high risk for work-related musculoskeletal disorders. Injury prevention among dancers has become challenging due to the dearth of research in the field. Therefore, a cross-sectional survey to determine the prevalence of musculoskeletal injuries among Sri Lankan traditional dancers is vital of need.<br>The main objective of this research was to evaluate the prevalence of common musculoskeletal injuries among university undergraduates who follow Sri Lankan traditional dancing. This study was a cross-sectional descriptive study that included undergraduate students from four local universities. Stratified sampling method was used to select 293 participants and an online questionnaire was used to collect data. Among the three types of traditional dancing styles, the majority were following Kandyan dancing: 45.1%. Out of the study sample, 190 dancers reported injuries with males indicating the highest rate of injuries. The most common injury types reported were strain and sprain. Kandyan dancers reported the highest number of injuries (p<0.025), contributing to the highest rate of injury due to strains (19.45%). Twirls and prolonged mandiya positions are found to be the common mechanisms that cause injuries. Only 10.6% of the participants approached physiotherapy treatments after an injury.<br>According to the findings of the current study, there is a significant rate of dancing-related injuries among Sri Lankan traditional dancers.
**Order of Authors:**	Surangika Imanthi Wadugodapitiya, Ph.D
	Journal PLOS ONE
	Geethika Chathurani, BSc
	Yasantha Bandara Dassanayake, BSc
	Sanduni Fernando, BSc
	Lahiru Gunarathna, BSc
	Lakshani Gunarathne, BSc
	Nadheera Chandrasekara, BSc
	Dilhari Senerath, MSc
**Additional Information:**	
**Question**	**Response**
**Financial Disclosure**<br><br>Enter a financial disclosure statement that	The author(s) received no specific funding for this work.

describes the sources of funding for the work included in this submission. Review the submission guidelines for detailed requirements. View published research articles from *PLOS ONE* for specific examples.<br><br>This statement is required for submission and **will appear in the published article** if the submission is accepted. Please make sure it is accurate.<br><br>**Unfunded studies**<br>Enter: *The author(s) received no specific funding for this work.*<br><br>**Funded studies**<br>Enter a statement with the following details:<br>• Initials of the authors who received each award<br>• Grant numbers awarded to each author<br>• The full name of each funder<br>• URL of each funder website<br>• Did the sponsors or funders play any role in the study design, data collection and analysis, decision to publish, or preparation of the manuscript?<br>• **NO** - Include this sentence at the end of your statement: *The funders had no role in study design, data collection and analysis, decision to publish, or preparation of the manuscript.*<br>• **YES** - Specify the role(s) played.<br><br>* typeset	
**Competing Interests**<br><br>Use the instructions below to enter a competing interest statement for this submission. On behalf of all authors, disclose any competing interests that could be perceived to bias this work—acknowledging all financial support and any other relevant financial or non-financial competing interests.<br><br>This statement is required for submission and **will appear in the published article** if the submission is accepted. Please make sure it is accurate and that any funding	The authors have declared that no competing interests exist.

sources listed in your Funding Information later in the submission form are also declared in your Financial Disclosure statement.<br><br>View published research articles from *PLOS ONE* for specific examples.<br><br>**NO authors have competing interests**<br><br>Enter: *The authors have declared that no competing interests exist.*<br><br>**Authors with competing interests**<br><br>Enter competing interest details beginning with this statement:<br><br>*I have read the journal's policy and the authors of this manuscript have the following competing interests: [insert competing interests here]*<br><br>* typeset	
**Ethics Statement**<br><br>Enter an ethics statement for this submission. This statement is required if the study involved:<br><br>• Human participants<br>• Human specimens or tissue<br>• Vertebrate animals or cephalopods<br>• Vertebrate embryos or tissues<br>• Field research<br><br>Write "N/A" if the submission does not require an ethics statement.<br><br>General guidance is provided below. Consult the submission guidelines for detailed instructions. **Make sure that all information entered here is included in the Methods section of the manuscript.**	Informed consent was obtained from all the participants in a written format that was included in the Google sheet. Ethical approval for the study was obtained from the Ethics Review Committee of the Faculty of Allied Health Sciences, University of Peradeniya, Sri Lanka<br>Approval no: AHS/ERC/2021/066

**Format for specific study types**<br><br>**Human Subject Research (involving human participants and/or tissue)**<br>• Give the name of the institutional review board or ethics committee that approved the study<br>• Include the approval number and/or a statement indicating approval of this research<br>• Indicate the form of consent obtained (written/oral) or the reason that consent was not obtained (e.g. the data were analyzed anonymously)<br><br>**Animal Research (involving vertebrate animals, embryos or tissues)**<br>• Provide the name of the Institutional Animal Care and Use Committee (IACUC) or other relevant ethics board that reviewed the study protocol, and indicate whether they approved this research or granted a formal waiver of ethical approval<br>• Include an approval number if one was obtained<br>• If the study involved *non-human primates*, add *additional details* about animal welfare and steps taken to ameliorate suffering<br>• If anesthesia, euthanasia, or any kind of animal sacrifice is part of the study, include briefly which substances and/or methods were applied<br><br>**Field Research**<br><br>Include the following details if this study involves the collection of plant, animal, or other materials from a natural setting:<br>• Field permit number<br>• Name of the institution or relevant body that granted permission	
**Data Availability**<br><br>Authors are required to make all data underlying the findings described fully available, without restriction, and from the time of publication. PLOS allows rare exceptions to address legal and ethical concerns. See the PLOS Data Policy and FAQ for detailed information.	Yes - all data are fully available without restriction

A Data Availability Statement describing where the data can be found is required at submission. Your answers to this question constitute the Data Availability Statement and **will be published in the article**, if accepted.<br><br>**Important:** Stating 'data available on request from the author' is not sufficient. If your data are only available upon request, select 'No' for the first question and explain your exceptional situation in the text box.<br><br>Do the authors confirm that all data underlying the findings described in their manuscript are fully available without restriction?	
**Describe where the data may be found in full sentences. If you are copying our sample text, replace any instances of XXX with the appropriate details.**<br><br>• If the data are **held or will be held in a public repository**, include URLs, accession numbers or DOIs. If this information will only be available after acceptance, indicate this by ticking the box below. For example: *All XXX files are available from the XXX database (accession number(s) XXX, XXX.).*<br>• If the data are all contained **within the manuscript and/or Supporting Information files**, enter the following: *All relevant data are within the manuscript and its Supporting Information files.*<br>• If neither of these applies but you are able to provide **details of access elsewhere**, with or without limitations, please do so. For example:<br><br>*Data cannot be shared publicly because of [XXX]. Data are available from the XXX Institutional Data Access / Ethics Committee (contact via XXX) for researchers who meet the criteria for access to confidential data.*<br><br>*The data underlying the results presented in the study are available from (include the name of the third party*	All relevant data are within the manuscript and its Supporting Information files.

and contact information or URL).<br>• This text is appropriate if the data are owned by a third party and authors do not have permission to share the data.<br><br>* typeset	
Additional data availability information:	

Manuscript

Click here to access/download;Manuscript;Manuscript_S Wadugodapitiya with citations and references.docx

Prevalence of Musculoskeletal Injuries among University Undergraduates Following Sri Lankan Traditional Dancing

Geethika Chathurani[1], Yasantha B. Dassanayake[1], Sanduni N. Fernando[1], Lahiru S. Gunarathna[1], Lakshani K. Gunarathne[1], Nadheera C. Chandrasekara[1], Dilhari Senarath[1], Surangika I. Wadugodapitiya[1]*

[1]Department of Physiotherapy, Faculty of Allied Health Sciences, University of Peradeniya, Peradeniya, Sri Lanka

*Corresponding author

Email: surangikaw@ahs.pdn.ac.lk

Abstract

Dancing is a demanding form of art that consists of a purposefully selected series of human movements presented in a rhythmic way. However, Dancers represent a medically under-served occupational group who are at high risk for work-related musculoskeletal disorders. Injury prevention among dancers has become challenging due to the dearth of research in the field. Therefore, a cross-sectional survey to determine the prevalence of musculoskeletal injuries among Sri Lankan traditional dancers is vital of need.

The main objective of this research was to evaluate the prevalence of common musculoskeletal injuries among university undergraduates who follow Sri Lankan traditional dancing. This study was a cross-sectional descriptive study that included undergraduate students from four local universities. Stratified sampling method was used to select 293 participants and an online questionnaire was used to collect data.

Among the three types of traditional dancing styles, the majority were following Kandyan dancing: 45.1%. Out of the study sample, 190 dancers reported injuries with males indicating the highest rate of injuries. The most common injury types reported were strain and sprain. Kandyan dancers reported the highest number of injuries ($p<0.025$), contributing to the highest rate of injury due to strains (19.45%). Twirls and prolonged *mandiya* positions are found to be the common mechanisms that cause injuries. Only 10.6% of the participants approached physiotherapy treatments after an injury.

According to the findings of the current study, there is a significant rate of dancing-related injuries among Sri Lankan traditional dancers.

Key words: injury mechanism, musculoskeletal injuries, prevalence, Sri Lankan traditional dancing, university undergraduates

Introduction

Dancing is a challenging form of art that consists of a rhythmically presented succession of purposefully selected series of human movements. Dancing necessitates a lot of practice, balance, motor coordination, and extremes of joint range of motion.

There are various types of traditional dancing styles such as ballet, Bharatanatyam, ballroom, contemporary, hip hop, jazz, tap dance, folk dance, Irish dance, modern dance, and swing dance. A dance form is designed with elements of traditional culture such as music, literature, and legends [1]. The origin of traditional dancing in Sri Lanka goes back to immemorial times of native tribes: '*Yakkas*', thus, imposing a paramount importance in Sri Lankan fine arts. There are three main styles in Sri Lankan traditional dancing: 'Kandyan dancing' or '*Udarata Natum*' of the hill country origin, 'Low country dancing' or '*Pahatharata Natum*' of the southern plains origin, and '*Sabaragamuwa* dancing' which originated in *Sabaragamuwa* province [1].

There are some variations in techniques and skills in these three traditional dance styles according to the teachers and different schools (Gurus). Backflips, acrobatics, and leaps are the main skills that are used in the Kandyan dancing style. The famous pirouettes (a twirl on one foot) are the highlights of the performance [2]. Though there are small variations in characteristics and poses of these dancing styles, '*Mandiya'* is the most fundamental position of all (figure 1). This position is achieved by placing the feet three steps away from each other, hips are flexed and abducted, knees are flexed and externally rotated, shoulders are retracted, while the chest is moved forward with arms at the shoulder level. The upper limbs are kept in shoulder abduction, elbow flexion, wrist, and fingers extension. However, the low country dancers bend the trunk more forward and move the feet more outward during the *mandiya* position, than the other two styles while *Sabaragamuwa* dancers use greater hip and knee flexion (figure 1). In addition, *Sabaragamuwa* dancers practically reach the floor without bending their backs and therefore, *Sabaragamuwa* dancing style requires extensive training.

[**Fig 1** near here]

Fig 1. *Mandiya* position of the three traditional dancing styles in Sri Lanka. From left to right, Kandyan dancing style, low country Dancing style, and *Sabaragamu* dancing style, respectively.

Traditional dancers are considered athletes since they perform complex, physically demanding postures, are required to train for extended periods of time and do not have seasonal breaks like for other sports [3]. However, dancers are usually a medically neglected occupational group with a high risk of work-related musculoskeletal disorders (WMSD) [4]. Therefore, the health concerns of traditional dancers are worthy of attention for several reasons because most dancers begin their training at an early age, which poses a significant impact on their future health status [5].

Injury prevention in dancers has become challenging due to lack of research and documentation in the field. Warm-up, cool down and flexibility exercises that are used in traditional dancing as well as approaches followed when returning to dancing after an injury should be carefully studied to minimize the injury rates. Although several studies have been carried out on typical musculoskeletal disorders associated with many traditional dancing forms, no research has been done on dance related injuries in Sri Lanka. Since there are three main types of traditional dancing in Sri Lanka, the injury prevalence, injury mechanism, and the site of injury may vary with regard to the style. Therefore, a cross sectional survey to identify the musculoskeletal injuries prevailing among Sri Lankan traditional dancers was proposed in this study.

Undergraduates who follow Sri Lankan traditional dancing were taken as the study sample in the present study because they show less age-related physiological changes and the young can be considered as healthier since they have minimal health problems. In addition, previous studies have revealed reasons such as dance related injuries, psychological and physiological stress have brought out a negative impact on the quality of life of younger dancers [6]. At present, traditional

dancing is taught in a number of dance schools and academies in Sri Lanka with variations in dancing techniques and exercises they follow. In comparison, the university undergraduates follow a similar curriculae and use almost identical dance methods, making them a better population to assess. Thus, this study focused on evaluating the prevalence of common musculoskeletal injuries among university undergraduates following Sri Lankan traditional dancing styles.

Materials and methods

Participants

A cross-sectional descriptive study was carried out among the undergraduates of Visual Arts & Design and Performing Arts Unit of the Department of Fine Arts, University of Kelaniya, the Department of Languages, Cultural Studies and Performing Arts, University of Sri Jayawardenapura, the Department of Performing Arts of Sri Palee Campus, University of Colombo and University of the Visual and Performing Arts, Sri Lanka, the four Universities which offer traditional dancing studies in their curriculum.

Both male and female undergraduates between the ages of 18 and 30 who follow one of the three types of Sri Lankan traditional dancing styles as their major subject were recruited. The students who follow other dancing types together with the traditional dancing, those who engage in dancing for less than four years, who do not engage in regular dancing practices, engage in sports activities, have a history of musculoskeletal problems and injuries from sources other than dancing, who have undergone surgeries in the upper and lower limbs, who have deformities and congenital abnormalities in the upper or lower limbs and in the spine were excluded from the study. The sample size for the study was 293 which was calculated using Solvin's formula [7]. The stratified sampling technique was used to select the participants for the study.

Study procedure

A standard self-administered questionnaire used for assessing musculoskeletal injuries in ballet dancers that included four components related to dancing: socio-demographic data, dance related

factors, injury related factors and treatment options was provided to the sample in the form of a Google sheet [3]. From the returned questionnaires, 293 participants who fulfill the inclusion criteria were selected randomly. Informed consent was obtained from all the participants in a written format that was included in the Google sheet. Ethical approval for the study was obtained from the Ethics Review Committee of the Faculty of Allied Health Sciences, University of Peradeniya, Sri Lanka. Also, confidentiality of the participants and the information collected was assured.

Data analysis

Descriptive data was presented using absolute and relative frequencies of categorical variables such as injury prevalence, type, location and mechanism of injury and data were categorized with relevance to the three traditional dancing types and gender. Continuous variables such as body mass index (BMI), age, experience in dancing, duration of practice and intervals in one session were presented as Mean ± Standard Deviation. Association between dancing experience, duration of one practice session and the number of intervals in one dancing session with main traditional dancing style and gender of the participants was identified using One-way ANOVA test while the association between stretching exercises and practicing speed with main traditional dancing style, gender and the academic year of the participants was identified using Chi-square test. Dependency of injury prevalence and related factors on the Sri Lankan traditional dancing styles and gender was also identified using Chi-square test. All statistical analyses were conducted using SPSS statistical software, version 25 (SPSS, IBM Corp. Armonk, NY, USA).

Results

Characteristics of the participants

The mean age of the participants was 23 (1.6) years and the majority was females at 64.5%. Among the three types of traditional dancing styles, a majority of 45.1% follows Kandyan dancing. The mean BMI of the participants is 22.34 (3.6) kg/m^2 (Table 1).

Table 1. Characteristics of the participants (n=293)

Variables	No of participants	Percentage (%)
Age<br>23 (1.6) years	-	-
Gender		
Male	104	35.5
Female	189	64.5
Main dancing style		
Kandyan	132	45.1
Low country	92	31.4
Sabaragamu	69	23.5
BMI<br>22.34 (3.6) kg/m^2	-	-

Association between dancing-related factors with Sri Lankan traditional dancing styles and the gender of the participants

The results have shown that experience in dancing, the number of hours practiced per day, the number of days practiced per week, intervals taken within one dancing session have a significant association with the dancing style and the gender of the participants (Table 2).

Table 2. Association between dancing-related factors with the main traditional dancing style and gender of the participants

Variables	Main traditional dancing style					Gender		
	Kandyan	Low country	Sabaragamu	*P* value	Effect size	Male	Female	*P* Value
	Mean (SD)					Mean (SD)		
Dancing experience (years)	14.78 (3.66)	13.34 (3.68)	12.09 (3.45)	0.000[a]		12.35 (3.49)	14.44 (3.71)	0.000[a]
Practice hours per day	4.06 (2.65)	5.37 (2.05)	5.44 (2.03)	0.000[a]		5.16 (2.18)	4.41 (2.47)	0.284[a]
Practice days per week	3.89 (1.52)	4.71 (1.05)	4.77 (0.94)	0.002[a]		4.56 (1.17)	4.24 (1.39)	0.047[a]
Intervals in one dancing session (hours)	2.2 (0.62)	2.02 (0.59)	1.49 (0.78)	0.004[a]		1.43 (0.76)	2.2 (0.56)	0.000[a]
	***n* (%)**					***n* (%)**		
Stretching exercise + Warm up exercise	103 (35.2)	65 (22.2)	49 (16.7)	0.373[b]	0.022	71 (24.23)	146 (49.82)	0.093[b]

Stretching exercise + Cool down exercise		38 (12.9)	24 (8.2)	15 (5.1)	0.559[b]	0.032	24 (8.19)	53 (18.08)	0.355[b]
Practicing speed	High	7 (2.3)	4 (1.36)	4 (1.36)	0.916[b]	0.053	10 (3.41)	5 (1.7)	0.013[b]
	Moderate	122 (41.6)	84 (28.66)	63 (21.5)			89 (30.37)	180 (61.43)	
	Low	3 (1.02)	4 (1.36)	2 (0.68)			5 (1.7)	4 (1.36)	
Dancing floor	Cement floor	91 (31.05)	73 (24.91)	55 (18.77)	0.144[b]	0.008	81 (27.64)	138 (47.09)	0.665[b]
	Tile floor	33 (11.26)	19 (6.48)	13 (4.43)			21 (7.16)	44 (15.01)	
	Sand floor	4 (1.36)	0 (0)	0 (0)			2 (0.68)	2 (0.68)	

Data shown as Mean (SD) or *n* (%) with 95% confidence interval. [a]One-way ANOVA. [b]Chi-square test

Dependency of injury prevalence and related factors on the Sri Lankan traditional dancing styles and gender

In the study sample, 190 dancers reported injuries in dancing. According to the data, strain was the highest reported injury type with 31.04%, and the second highest was sprain (17.7%). Subluxation, fractures and other injuries reported were 8.9%, 1.7% and 16.4%, respectively. The highest rate of injuries by strain (19.45%) was reported by students who follow Kandyan Dancing whereas; sprain was more common among Low country and Sabaragamu dancers.

When the prevalence of injuries in each dancing style was assessed, strains, subluxations, sprains, and other injuries showed significant associations with the type of traditional dancing style. Males reported a substantially higher overall injury rate than that of females according to the prevalence of injuries with relevance to the gender. Further, sprain is the most common type of injury in males, while strain is the most common in females (Table 3).

Table 3. Association between injury prevalence and related factors with the main traditional dancing style and the gender of the participants

**Variables**	**Main traditional dancing style**					**Gender**		
	**Kandyan**	**Low country**	**Sabaragamu**	***P* – value**	**Effect size**	**Male**	**Female**	***P* - value**

Injuries during dancing n=190 *n* (%)	96 (32.76%)	51 (17.40%)	43 (14.67%)	0.025[b]	0.025	82 (27.98%)	108 (36.86%)	0.000[b]
**Type of injuries *n* (%)**								
Fracture n=5	1 (0.34%)	2 (0.68%)	2 (0.68%)	0.493[b]	0.02	3 (1.02%)	2 (0.68%)	0.248[b]
Strain n=91	57 (19.45%)	21 (7.16%)	13 (4.43%)	0.000[b]	0	36 (12.28%)	55 (18.77%)	0.329[b]
Subluxation n=26	7 (2.38%)	8 (2.73%)	11 (3.75%)	0.042[b]	0.002	17 (5.8%)	9 (3.07%)	0.001[b]
Sprain n=52	15 (5.11%)	19 (6.48%)	18 6.14%)	0.023[b]	0.001	32 (10.92%)	20 (6.82%)	0.000[b]
Other injuries n=48	30 (10.23%)	10 (3.41%)	8 (2.73%)	0.029[b]	0.001	18 (6.14%)	30 (10.25%)	0.751[b]

Data shown as *n* (%) with 95% confidence interval. [b]Chi-square test

Association between the mechanism of injury, location of the injury and the Sri Lankan traditional dancing style and the gender

Twirls and prolonged *mandiya* positions were the common mechanisms that cause a higher injury rate among dancers. Nevertheless, prolonged *mandiya* position, jumps and other combined movements were found to have a potent correlation with the higher injury rate reported among Kandyan dancers while backflips, acrobatics, and twirls were the movements causing more injuries among Low country dancers. Prolonged *mandiya* position, backflips and acrobatics cause significantly higher injury rates in males compared to that in females. Females reported a higher injury rate in neck, shoulder, upper chest, elbow, hand, thigh, knee, and leg than males. However, ankle, knee, and thigh are the most common injury sites reported by the traditional dancers regardless of the type of dancing style and gender. Further, neck, lumbar spine, thigh, knee and ankle were the most prevalent injury sites among Kandyan dancers (Table 4).

Table 4. Association between the mechanism of injury and location of injury with the main traditional dancing style and the gender of the participants

Variables	Main traditional dancing style					Gender		
	Kandyan	Low country	Sabaragamu	*P* – value	Effect size	Male	Female	*P* value
	**Mechanism of injury *n* (%)**							
Prolong mandiya position n=43	20 (6.8)	5 (1.7)	18 (6.1)	0.001[b]	0	28 (9.55)	15 (5.1)	0.000[b]
Back flips n=25	7 (2.3)	11 (3.75)	7(2.3)	0.185[b]	0.01	20 (6.82)	5 (1.7)	0.000[b]
Acrobatics n=25	7 (2.3)	11 (3.75)	7 (2.3)	0.185[b]	0.01	20 6.82)	5 (1.7)	0.000[b]
Leaps n=24	8 (2.7)	8 (2.7)	8 (2.7)	0.389[b]	0.022	10 (3.41)	14 (4.7)	0.510[b]
Twirls n=46	14 (4.77)	23 (7.8)	9 (3.07)	0.011[b]	0.0006	20 (6.82)	26 (8.87)	0.218[b]
Jumps n=29	24 (8.19)	2 (0.68)	3 (1.02)	0.000[b]	0	7 (2.38)	22 (7.5)	0.178[b]
Other mechanisms n=72	49 (16.7)	16 (5.4)	7 (2.3)	0.000[b]	0	22 (7.5)	50 (17.06)	0.313[b]
	**Location of injury *n* (%)**							
Face n=0	0	0	0	.[b]	-	0	0	. [b]
Neck n=14	12 (4.09)	2 (0.68)	0	0.006[b]	0.0003	4 (1.36)	10 (3.41)	0.579[b]
Shoulder and upper chest n=21	9 (3.07)	10 (3.4)	2 (0.68)	0.149[b]	0.008	10 (3.41)	11 (3.75)	0.228[b]
Elbow n=11	6 (2.04)	4 (1.36)	1 (0.34)	0.514[b]	0.030	3 (1.03)	8 (2.73)	0.561[b]
Forearm n=4	2 (0.68)	1 (0.34)	1 (0.34)	0.961[b]	0.056	2 (0.68)	2 (0.68)	0.542[b]
Hand n=5	2 (0.68)	3 (1.02)	0(0)	0.279[b]	0.016	0	5 (1.7)	0.094[b]
Lumbar n=36	20 (6.8)	9 (3.07)	7 (2.3)	0.400[b]	0.023	18 (6.14)	18 (6.14)	0.052[b]
Pelvic n=0	0	0	0	. [b]	-	0	0	. [b]
Buttocks n=12	8 (2.7)	2 (0.68)	2 (0.68)	0.299[b]	0.017	7 (2.38)	5 (1.7)	0.091[b]
Thigh n=51	30 (10.23)	9 (3.07)	12 (4.09)	0.042[b]	0.002	25 (8.53)	26 (8.87)	0.026[b]
Knee n=52	29 (9.89)	12 (4.09)	11 (3.75)	0.206[b]	0.012	19 (6.48)	33 (11.2)	0.862[b]
Leg n=24	15 (5.11)	7 (2.3)	2 (0.68)	0.112[b]	0.0065	8 (2.73)	16 (5.46)	0.817[b]
Ankle n=55	21 (7.16)	19 (6.48)	15 (5.11)	0.517[b]	0.03	28 (9.55)	27 (9.21)	0.008[b]
Foot n=0	0	0	0	. [b]	-	0	0	. [b]
Abdomen n=0	0	0	0	. [b]	-	0	0	. [b]

Data shown *n* (%) with 95% confidence interval. [a]One-way ANOVA. [b]Chi-square test

Dancers' approach to treatment options following an injury

Only 16.3% of the participants had received Physiotherapy treatments after an injury, while 63.15% opted for other types of care.

Discussion

Traditional forms of dancing in Sri Lanka are high-performance physical activities that require an advanced level of technical skills. These dancers frequently place great stress on tendons, muscles, bones, and joints. Therefore, they are a high-risk group for chronic injuries. Though there are studies explaining common musculoskeletal problems associated with various traditional dancing types, no research has been carried out in Sri Lanka assessing the traditional dancers. Thus, this study focused on assessing the prevalence of musculoskeletal injuries among university undergraduates who follow Sri Lankan traditional dancing.

According to the study results, the majority of dancers practiced warm-up exercises and only 20% of dancers followed cool-down exercises after a dancing session. Although the importance of warm-up and cool-down in general exercise and sports is widely acknowledged, awareness of their importance among Sri Lankan traditional dancers is poor. Even though the majority of dancers regularly performed the warm-up exercises, musculoskeletal pain was widely reported among them which highlights the fact that musculotendinous injury does not purely result from lack of elasticity but when tension demands of the muscle exceeds the tension generating capability of the muscle [8].

In the current study, dance-related injuries were reported by 64.8% of the participants out of which strain and sprain injuries were the most common injuries while fractures were less common. This could be due to the fact that all three styles of dancing employ extreme motions that can injure ligaments and muscles. Despite dancing being an athletic exercise, fractures are uncommon among dancers as they maintain a lower contact force [9]. Soft tissues, on the other hand, are subjected to prolonged stress during dancing which may be a reason for the higher rate

of sprains and strains among dancers [10]. Similarly, a study conducted in Korea reported that the prevalence of strains and sprains was as high as 89.6% among breakdancers [11].

In this study, back, knee and ankle were the most frequently reported sites of injuries. These findings are in line with recent studies conducted in India to assess dance related injuries in Barathanatyam dancing style [5,12,13]. Similarly, a systematic review on epidemiology on musculoskeletal injuries in Indian classical dancers revealed a higher incidence of back injuries followed by injuries to the knee and ankle in all forms of classical dancing [14]. Apart from Indian classical dancing, dancing styles such as Irish, Flamenco, Turkish, and Morris dance from Great Britain reported a higher incidence of lower limb injuries [15]. In addition, the current study found that injuries in neck, thigh, knee, and ankle are prevalent among Kandyan dancers with a significantly higher percentages of neck and thigh injuries. Further, excessive head movements cause strain on neck muscles due to extreme movements of spine when arching the head or neck [16]. Furthermore, it was found that ankle is the most common injury site among Low country and *Sabaragamu* dancers. Other studies show that Ballet dancers also found to have more injuries in the spine, shoulder, ankle, and foot [3,17]. Likewise, modern dancers reported the same areas as the sites which are more prone for injuries including lower back and foot [18,19]. The ankle has been identified as a common site for injuries in many of these studies as the body weight shifts to the ground via the ankle joint, and ankle is subjected to sudden torsions while dancing [20,21]. Stretching exercises and active range of motion exercises through the entire joint play should be encouraged before dance sessions to enhance the flexibility of the muscles around the ankle and other areas reported to be more susceptible to injuries. However, in dance styles like Pole dance and Belly dance, the most common injury sites are the shoulder, wrist and pelvic area, respectively [15,22]. Although the lower limb is the most prevalent injury site in most dance genres, it varies depending on the dancing movements used in each dancing style.

In the current study, it was evident that as vigorous, high-speed movements like twirls and prolonged *mandiya* position are more frequently used in Kandyan form of dancing where the injury

rate is higher compared to Low country and *Sabaragamu* dancers. Low country dancers, on the other hand, sustain fewer injuries than the other two categories of dancers owing to the slower pace movements and performing more warm-up exercises than *Sabaragamuwa* and Kandyan dancers. A report published on the importance of warm-up and cool down exercises as well verified that the risk of injuries while dancing were lowered with effective warm-up exercises [23].

In traditional Sri Lankan styles, only the highly experienced dancers use mechanisms like jumping, back flips, acrobatics and leaps while majority use *mandiya* position and twirls [5]. According to the results of the study, twirls and prolonged *mandiya* position were found to be the common movements that cause higher rate of injuries among traditional dancers in Sri Lanka. Twirls caused majority of injuries in low country dancers as it is a basic movement of the style. On the contrary, *Sabaragamuwa* dancers suffer from injuries mostly as a result of prolonged *mandiya* position. Likewise, among Indian Baratantyam dancer "*Aramandi*" position which is similar to the prolonged *mandiya* position in Sri Lankan traditional dancing, has caused most of the injuries among Indian Baratanatyam dancers [5]. Prolonged movements overload the knee and ankle joints and results in micro traumas to the ligaments and muscles that surrounds them [3]. The current study clearly shows that holding of prolonged positions during dancing cause greater rate of injuries in Sri Lankan traditional dancing, with the knee and ankle joints being the most vulnerable injury sites. In addition, both the literature and the current study show that repetitive and vigorous movements results in injuries [3]. However, the mechanism of dance related injuries is different among Ballet dancers where jumps, lifting and pointe work are the common movement patterns [24]. Hence, previous studies and the current research suggest the mechanism of injury varies depending on the dancing style.

The present study revealed that males experience a higher rate of injuries during dancing which might have arisen from factors such as lack of experience than females, using more vigorous movements, performing fewer warm-up and cool-down exercises, lengthy practice sessions per day than females, having brief breaks between two dancing sessions, and practicing at a faster pace

than female dancers. These findings are compatible with the studies conducted on Ballet dancers and Ballroom dancers that found high injury rate among the male dancers [24.25].

According to the literature, dancers who dance on hard flooring are more likely to get injuried [9,26]. A biomechanical study comparing three different surfaces used by Indian Bharathnatyam dancers found that non reliance surfaces like concrete produce higher impact forces while dancing and it was mentioned that the type of the dancing floor is an important factor to be considered in order to minimize dance related injuries [27]. The current study also found that dancers who perform on cement floors are more prone for injuries.

Physiotherapy treatment is the optimum approach for musculoskeletal injuries. However, according to the results of the current study, only 16.3% of the injured dancers had undergone physiotherapy treatments. In addition, according to studies that reported dance related injuries dancers felt compulsive to return to dance before their injuries were healed as they had invested considerable amount of time in dancing [5]. Further, when the injured ligament or muscle does not receive adequate rest and appropriate exercise there is a frequent recurrence of an injury. Thus, dancing while having biomechanical dysfunction or muscle imbalance can lead to severe consequences that require the cessation of dance activities, which in turn increase the stress levels of dancers [5,28].

The present study had certain limitations. First, due to the COVID-19 pandemic, data was collected via a Google sheet which might have hindered the true responses of the participants. Further, for the past two years all of the study participants have had less frequent chances to perform and practice dancing. Therefore, it may have been difficult for them to recall their memory on dancing related injuries.

Conclusion

According to the findings of the current study, there is a significant rate of dancing-related injuries among Sri Lankan traditional dancers. Male dancers showed a higher rate of injuries

than female dancers; while Kandyan dancers had the highest injury rate. Most of the dancers have suffered from soft tissue injuries like strains and sprains that are prevalent in other dancing styles in the world. The thigh and knee are the most common injury sites among Sri Lankan traditional dancers. In addition, this study has identified that only a few dancers approached physiotherapy treatments following an injury. Moreover this study paved the way for a number of further studies aimed at reducing dance-related injuries and enhancing the career and quality of life of Sri Lankan traditional dancers. The identification of various dancing-related factors that cause injuries in Sri Lankan traditional dancing, such as the type of common injuries, most vulnerable sites for injuries, and the movement patterns that cause higher injury rates, may help to improve therapeutic actions aimed at the proper biomechanics and improved performance.

Supporting information

Questionnaire

Acknowledgements

We thank Prabodani Samarakoon, Instructor in English, University of Peradeniya, Sri Lanka for her assistance in proof reading and language editing.

Disclosure of interest

The authors report no conflict of interest

Funding

The authors received no financial support for the research, authorship, and/or publication of this article.

References

1. Karunaratne PVM. Art and tradition of Sri Lanka. S. Godage and Brothers (Pvt) Ltd n.d;11. doi:dl.lib.mrt.ac.lk/handle/123/14437

2. Dissanayake M. Sinhala narthana kalawa. Godage International Publisher. 2009.
3. Costa MSS, Ferreira AS, Orsini M, Silva EB, Felicio LR. Characteristics and prevalence of musculoskeletal injury in professional and non-professional ballet dancers. Braz J Phys Ther 2016;20(2):166–175. https://doi.org/10.1590/bjpt-rbf.2014.0142
4. Bronner S, Ojofeitimi S, Spriggs J. Occupational musculoskeletal disorders in dancers. Physical therapy reviews. 2003;8(2):57–68. https://doi.org/10.1179/108331903225002416
5. Nair SP, Kotian S, Hiller C, Mullerpatan R. Survey of Musculoskeletal Disorders Among Indian Dancers in Mumbai and Mangalore. Journal of Dance Medicine & Science : Official Publication of the International Association for Dance Medicine & Science. 2018;22(2):67–74. https://doi.org/10.12678/1089-313X.22.2.67
6. Krasnow D, Mainwaring L, Kerr G. Injury, stress, and perfectionism in young dancers and gymnasts. Journal of Dance Medicine & Science. 1999;3(2):51–58.
7. Habib A, Johargy A, Mahmood K, Humma H. Design and Determination of The Sample Size In Medical Research. IOSR Journal of Dental and Medical Sciences. 2014;13(5):21–31. doi.org/10.9790/0853-13562131
8. Thomas H, Tarr J. Dancers' perceptions of pain and injury. Journal of Dance Medicine & Science : Official Publication of the International Association for Dance Medicine & Science. 2009;13(2):51–59. PMID: 19508809
9. Russell J. Preventing dance injuries: current perspectives. Open Access Journal of Sports Medicine. 2013:199. https://doi.org/10.2147/oajsm.s36529

10. Sohl P, Bowling A. Injuries to dancers: Prevalence, treatment and prevention. Sports medicine (Auckland, N.Z.). 1990;9(5):317–322. https://doi.org/10.2165/00007256-199009050-00006

11. Cho CH, Song KS, Min BW, Lee SM, Chang HW, Eum DS. Musculoskeletal injuries in break-dancers. Injury. 2009;40(11):1207–1211. https://doi.org/10.1016/j.injury.2009.05.019

12. Kumar LP, Shenoy SJ. Survey of Musculoskeletal Injuries among Female Bharatanatyam Dancers in the Udupi District of India. Med Probl Perform Art. 2021;36(3):199-206. doi:10.21091/mppa.2021.3022

13. Nair SP, Kotian S, Hiller C, Mullerpatan R. Survey of Musculoskeletal Disorders Among Indian Dancers in Mumbai and Mangalore. J Dance Med Sci. 2018;22(2):67-74. doi:10.12678/1089-313X.22.2.67

14. Masal S and Borkar P. Epidemiology of musculoskeletal injuries in Indian classical dancers: A systematic review. Int J of Phy Edu, Sports, and Health. 2021;8(3):310-319.

15. Taboada-Iglesias Y, Abalo-Nunez R, Garcia-Remeseiro T. Traditional Dances and their Characteristic Injury Profiles. Systematic Review. Apunts Education Fisica y Deportes. 2020;141:1-10. http://doi.org/10.5672/apunts.2014-0983.es.(2020/3).141.01

16. McCabe TR, Ty Hopkins J, Vehrs P, Draper DO. Contributions of muscle fatigue to a neuromuscular neck injury in female ballroom dancers. Medical Problems of Performing Artists. 2013;28(2):84–90. https://doi.org/10.21091/mppa.2013.2015

17. Tsekoura M, Billis E, Panopoulou K, Fousekis K, Tsepis E. Musculoskeletal Disorders among Greek Professional Ballet Dancers. British J of Med and Med Research. 2017;19(11):1–8. https://doi.org/10.9734/BJMMR/2017/31876

18. Shah S, Weiss DS, Burchette RJ. Injuries in Professional Modern Dancers. Journal of Dance Medicine and Science. 2012;16(1):17–25. PMID: 22390950
19. Van Winden DPAM, Van Rijn RM, Richardson A, Savelsbergh GJP, Oudejans RRD, Stubbe JH. Detailed injury epidemiology in contemporary dance: A 1-year prospective study of 134 students. BMJ Open Sport and Exercise Medicine. 2019;5(1):1–5. https://doi.org/10.1136/bmjsem-2018-000453
20. Wiesler ER, Hunter DM, Martin DF, Curl WW, Hoen H. Ankle flexibility and injury patterns in dancers. The American journal of sports medicine. 1996;24(6):754–757. https://doi.org/10.1177/036354659602400609
21. Botsis AE, Schwarz NA, Harper ME, Liu W, Rooney C A, Gurchiek LR, Kovaleski JE. Effect of Kinesio® taping on ankle complex motion and stiffness and jump landing time to stabilization in female ballet dancers. Journal of Functional Morphology and Kinesiology. 2019;4(2).
https://doi.org/10.3390/jfmk4020019
22. Lee JY, Lin L, Tan A. Prevalence of pole dance injuries from a global online survey. J Sports Med Phys Fitness. 2020;60(2):270-275.
doi:10.23736/S0022-4707.19.09957-2
23. Fadzali F. The Importance of Warm Up & Cool Down. 2020.
24. Allen N, Nevill A, Brooks J, Koutedakis Y, Wyon M. Ballet injuries: Injury incidence and severity over 1 year. Journal of Orthopaedic and Sports Physical Therapy. 2012;42(9):781–790.
https://doi.org/10.2519/jospt.2012.3893
25. Cardoso AA, Reis NM, Vieira MCS, Borgatto AF, Folle A, Guimaraes ACA. Associated factors and profile of injuries in professional ballroom dancers in Brazil: a cross-sectional study. Motriz rev educ fis. 2020;26(2): e10200077.
https://doi.org/10.1590/s1980-6574202000020077

26. Pratap SS, Sona K, Ashok KS, Parag S. Prevalence of Musculoskeletal Injuries in Participants enrolled in Zumba-based Training. Journal of Musculoskeletal Research. 2020:S021895772050013X–. doi:10.1142/S021895772050013X

27. Anbarasi V, Ashraf M, Prakash H, Rajan DV. Vertical ground reaction force while performing an Indian classical dance, “Bharatanatyam”- A biomechanical study comparing three different surfaces. Int J of Ortho and Phys. 2020;2(1):01-04.

28. Rivera DC, Alexander JL, Nehrenz GM, Fields BJ, Circle ES. Dancers’ perceptions of injuries. Journal of Music and Dance. 2012;2(1):9–12. https://doi.org/10.5897/JMD11.006.

Figure

Click here to access/download;Figure;Fig 1.jpg

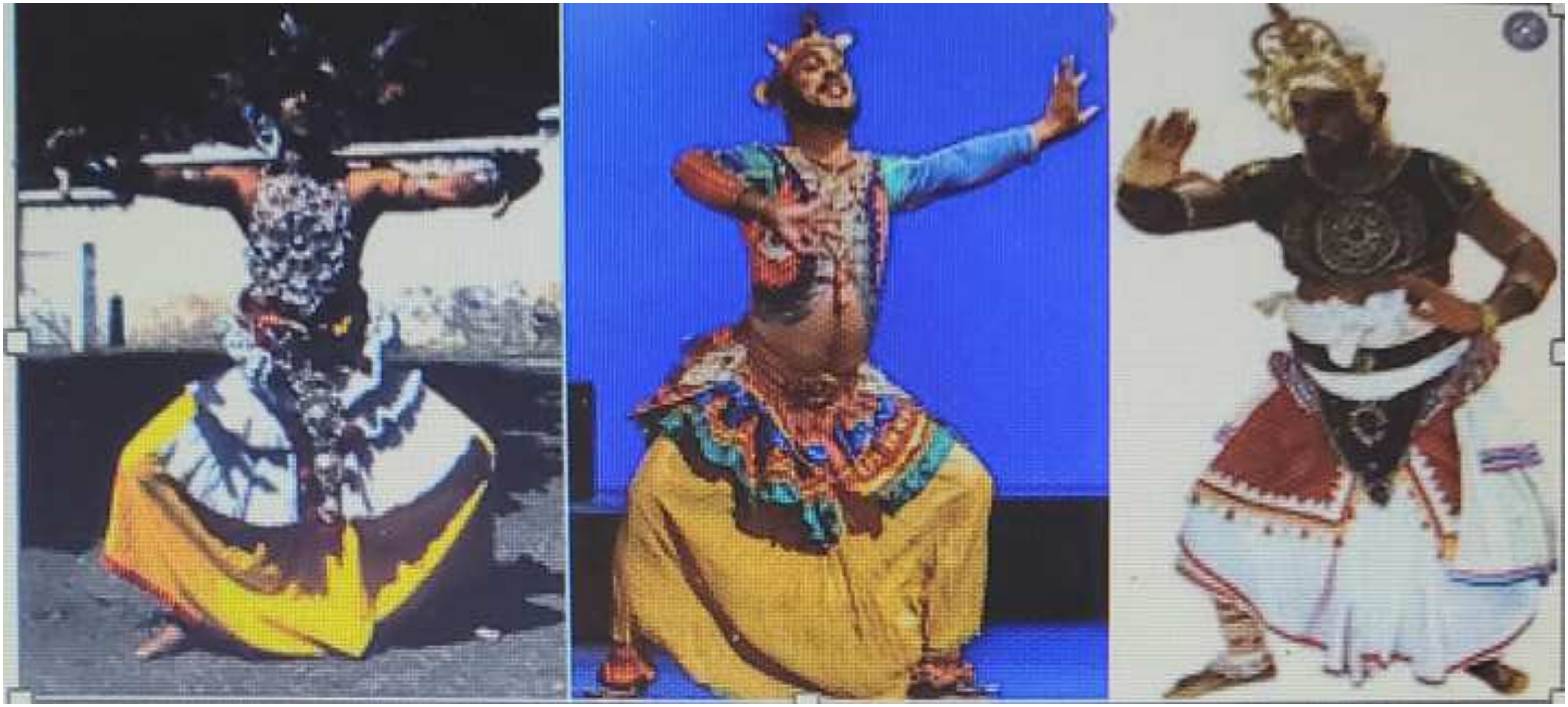

Click here to access/download
Supporting Information
Supporting information_questinnaire.docx
